# Fiber-Optic Sensor-Based Remote Acoustic Emission Measurement in a 1000 °C Environment

**DOI:** 10.3390/s17122908

**Published:** 2017-12-14

**Authors:** Fengming Yu, Yoji Okabe

**Affiliations:** Institute of Industrial Science, University of Tokyo, Tokyo 153-8505, Japan; okabey@iis.u-tokyo.ac.jp

**Keywords:** fiber-optic Bragg grating, high temperature, acoustic emission, guided wave, non-destructive test

## Abstract

Recently, the authors have proposed a remote acoustic emission (AE) measurement configuration using a sensitive fiber-optic Bragg grating (FBG) sensor. In the configuration, the FBG sensor was remotely bonded on a plate, and an optical fiber was used as the waveguide to propagate AE waves from the adhesive point to the sensor. The previous work (Yu et al., Smart Materials and Structures 25 (10), 105,033 (2016)) has clarified the sensing principle behind the special remote measurement system that enables accurate remote sensing of AE signals. Since the silica-glass optical fibers have a high heat-resistance exceeding 1000 °C, this work presents a preliminary high-temperature AE detection method by using the optical fiber-based ultrasonic waveguide to propagate the AE from a high-temperature environment to a room-temperature environment, in which the FBG sensor could function as the receiver of the guided wave. As a result, the novel measurement configuration successfully achieved highly sensitive and stable AE detection in an alumina plate at elevated temperatures in the 100 °C to 1000 °C range. Due to its good performance, this detection method will be potentially useful for the non-destructive testing that can be performed in high-temperature environments to evaluate the microscopic damage in heat-resistant materials.

## 1. Introduction

Advanced composites including carbon fiber reinforced plastic (CFRP) consist of almost 50% of the total weight of the B787 civil aircraft to lower its weight [1]. Successful applications of CFRP have prompted the use of composites in the civil aerospace industry for building fuel-efficient aircraft. Recently, ceramic matrix composites (CMCs) have shown growth potential as new civil aviation engine material due to their heat-resistant and weight-saving properties [2]. However, the fracture process in CMCs must be clarified, particularly in the high-temperature engine-operating environment because fractures in the material can be caused by accumulation of microscopic damages, which is difficult to experimentally observe and evaluate in extreme environments and poses a major safety risk for CMC-made engine systems. Hence, development of reliable composite materials requires the use of in situ non-destructive testing (NDT) technologies for damage evaluation in material testing of coupon specimen at high temperature in furnaces.

Acoustic emission (AE) detection is a potential NDT method [3]. AE is an elastic stress wave generated at the occurrence of damage in materials. Detection of the ultrasonic components included in the wave could be used to evaluate the microscopic damage in the material in real time at both room temperature and high temperature. However, AE detection has not yet been used as a standard NDT method under extreme environments. The main reason for this is that the commonly used Pb(Zr,Ti)O_3_ (PZT) AE sensors show poor performance at temperatures greater than 200 °C. The conventional approach for high-temperature AE detection employs metal rod waveguides for propagating AE from the test environment to a room temperature environment where the PZT sensors can successfully detect the guided AE. However, the waveguide distorts the waveform and drastically damps the amplitude of the AE signals.

Several special heat-resistant PZT elements have been developed for successful high-temperature ultrasonic detection [4] and AE detection [5,6] at elevated temperatures. However, those sensors show a limited frequency bandwidth. Furthermore, several unresolved problems related to the electronic connection and bonding, the electrode, and the sensor package also hinder the practical application of PZT-based elements in extreme environments [7].

**Figure 1:**
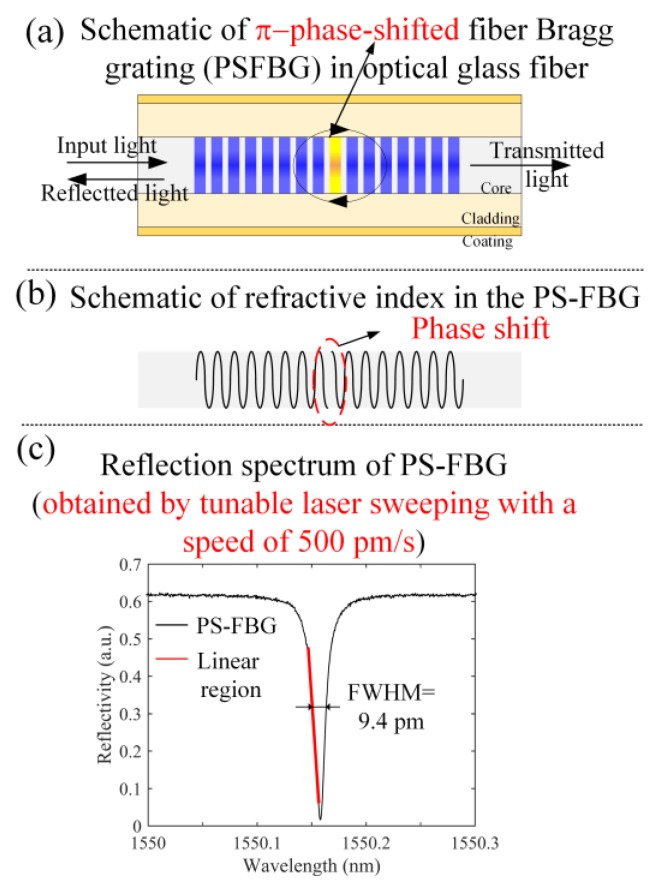
The introduction about PS-FBG.

On the other hand, most optical fiber sensors (OFSs) are inscribed in silica-glass fibers with high heat-resistance exceeding 1000 °C [8,9]. This makes the OFSs attractive for use in sensing devices operating at ultra-high temperatures [10]. Fiber Bragg gratings (FBGs) are a type of OFSs that have been used for structural health monitoring (SHM) of aerospace composites [11,12]. In particular, Wu and Okabe [13] developed a highly sensitive AE sensing system using a phase-shifted FBG (PSFBG). As shown in Figure 1a, a PS-FBG is produced by inserting a π phase shift into the middle of the grating area of an FBG. Due to the phase shift in the refractive index (Figure 1b), a sharp notch with FWHM of 9.4 pm is generated in the reflection spectrum (Figure 1c) to increase the sensitivity [13]. Meanwhile, a broader bandwidth response is achieved because of the short effective sensing length of a PS-FBG sensor [14].

The excellent performance of this system in AE detection enabled us to achieve in situ NDT in a CFRP composite [15,16,17]. However, thermal energy influences the conventional UV-induced (type I) FBGs, most of which completely disappeared at 900 °C [10], thus preventing the PSFBG sensors from operating at high temperatures.

To overcome the above mentioned problem in the PSFBG sensor and to achieve reliable high-temperature AE detection, we attempted to apply the adhesive method for remote measurement (ADRM) proposed in our previous research [18] to the PSFBG sensor-based AE detection. 

**Figure 2:**
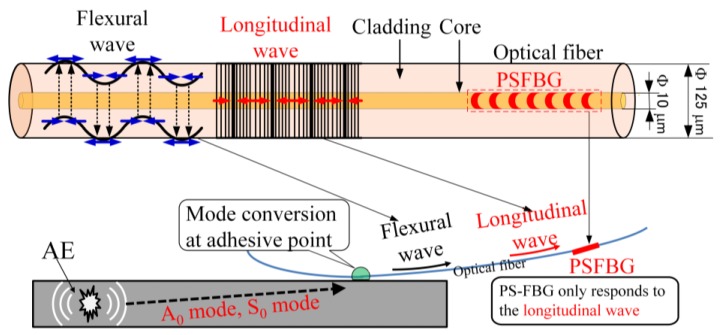
Detection mechanism of PS-FBG sensor in the ADRM configuration.

In the ADRM configuration, the PSFBG sensor was remotely bonded by an optical fiber, which was used as the waveguide to propagate AE wave from the adhesive point to the sensor. Unlike the metal waveguide used for the PZT-based AE detection at high temperature, the optical fiber-based waveguide allowed the PSFBG sensor to accurately respond to the original AE waveform at the adhesive point. Figure 2 illustrates how the PSFBG sensor receives the AE wave. AE propagates in the plate as a Lamb wave until its arrival at the adhesive point. Then, the Lamb wave transforms into the other type of wave propagating in the optical fiber that is an extremely thin cylinder. This wave includes a basic longitudinal wave with the axial strain component in the core of the optical fiber, and a transverse basic flexural wave consisting mainly of the shear strain component in the core. Due to the ideal wave propagation system provided by the thin optical fiber with a small diameter (125 µm, without polymer coating), the two modes propagated along the optical fiber without any mode transformation. Moreover, the ultrasonic damping in the optical fiber was small, thus enabling feasible remote AE measurements. In addition, the PSFBG sensor is located in the core (10 µm) of the glass fiber and only responds to the axial strain. As a result, the PSFBG sensor selectively received only the longitudinal wave and did not receive the transverse wave. Since wavelengths of S_0_ and A_0_ modes propagating in the plate were in the order of some centimeters that were much longer than the diameter of the optical fiber, the longitudinal mode, including the wave components, showed no dispersion in the optical fiber. Hence, the detected waveform could accurately reflect the original waveform of the A_0_ and S_0_ modes at the adhesive point. This sensing principle in the ADRM configuration also enabled the PSFBG sensor to remain highly sensitive to the ultrasonic wave over a broad bandwidth [13,18].

As mentioned previously, the silica-glass optical fiber shows a high heat resistance of over 1000 °C. Therefore, for a preliminary demonstration of the high-temperature AE detection method, we exploit the sensing characteristics of the ADRM configuration. In this method, we used the optical fiber waveguide to propagate AEs from the structural material in a high temperature environment to the room temperature environment where the PSFBG sensor could operate as the receiver of the guided AE wave. Experiments described in Section 4 verified that this approach protected the PSFBG sensor from exposure to the thermal energy, and displayed excellent performance for AE detection at elevated temperatures.

Prior to discussing the experimental verification, the characteristics of the optical fiber-based ultrasonic waveguide at high-temperature, and the coupling between the material and the optical fiber, which were crucial for the reliable performance of the ADRM configuration in the high-temperature AE detection, must be discussed. Hence, Section 2 describes the ultrasonic detection experiment performed to demonstrate the characteristics of the wave propagation system provided by the optical fiber at elevated temperatures. Additionally, the experiment also demonstrated that the outstanding durability of the ultrasonic waveguide allowed the FBG sensor to perform AE detection at over 1000 °C for several hours, or even longer. Then, using a 3-D finite element model (FEM), we examined how the changes in the adhesive material and adhesive length affected the detected waveforms in the ADRM configuration, as shown in Section 3.

## 2. Optical Fiber-Based Ultrasonic Waveguide in High-Temperature Environment

**Figure 3:**
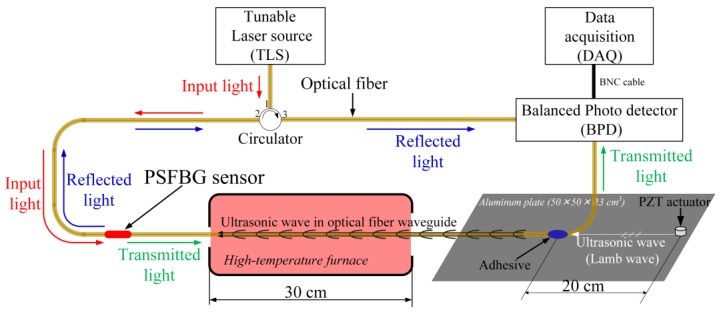
Ultrasonic detection using the optical fiber-waveguide in high temperature.

In Section 1, it was noted that the use of an optical fiber-based ultrasonic waveguide was an important contributing factor in the remote measurement method. Thus, successful high-temperature AE detection using the ADRM configuration required that the waveguide functioned stably, even in extreme environments. However, high temperatures of over 1000 °C may have changed the materials properties of the silica glass fiber, and, in turn, distorted the detected waveform. Hence, we conducted an ultrasonic detection experiment, shown in Figure 3, to verify whether thermal energy affected the wave propagation in the optical fiber.

In the experiment, the optical fiber was glued on an aluminum plate (length × width × thickness = 500 × 500 × 3 mm^3^) to remotely bond the PSFBG sensor to receive the ultrasonic wave excited by a PZT-type actuator (NF, AE900W) in the plate. A segment of the optical fiber between the adhesive point and PSFBG was passed through a high-temperature tubular furnace with a length of 30 cm. This experimental setup could protect other parts, including the PSFBG, adhesive, actuator, and plate from the exposure to thermal energy. Consequently, changing the temperature in the furnace only affected the optical fiber segment. 

The highly sensitive balanced sensing system [13] was used to demodulate the AE signal detected by the PSFBG sensor. The system uses the edge filter method to demodulate the shift of the Bragg wavelength due to the change in the strain applied to the PS-FBG sensor. The PS-FBG sensor separates the light from a tunable laser (TLS, Agilent, 81682A, Santa Clara, CA, USA) with laser linewidth of 100 kHz into a transmitted part and a reflected part. A balanced photo detector (BPD, New Focus, 2117, Irvine, CA, USA) converts the light’s power into an electrical signal. This method eliminates the DC component and doubles the AC component. In addition, the balanced photo detector removes noise in the light source intensity due to the difference in the power of the two lights. As a result, PS-FBG sensor in this sensing system is much more sensitive than a normal FBG sensor.

**Figure 4:**
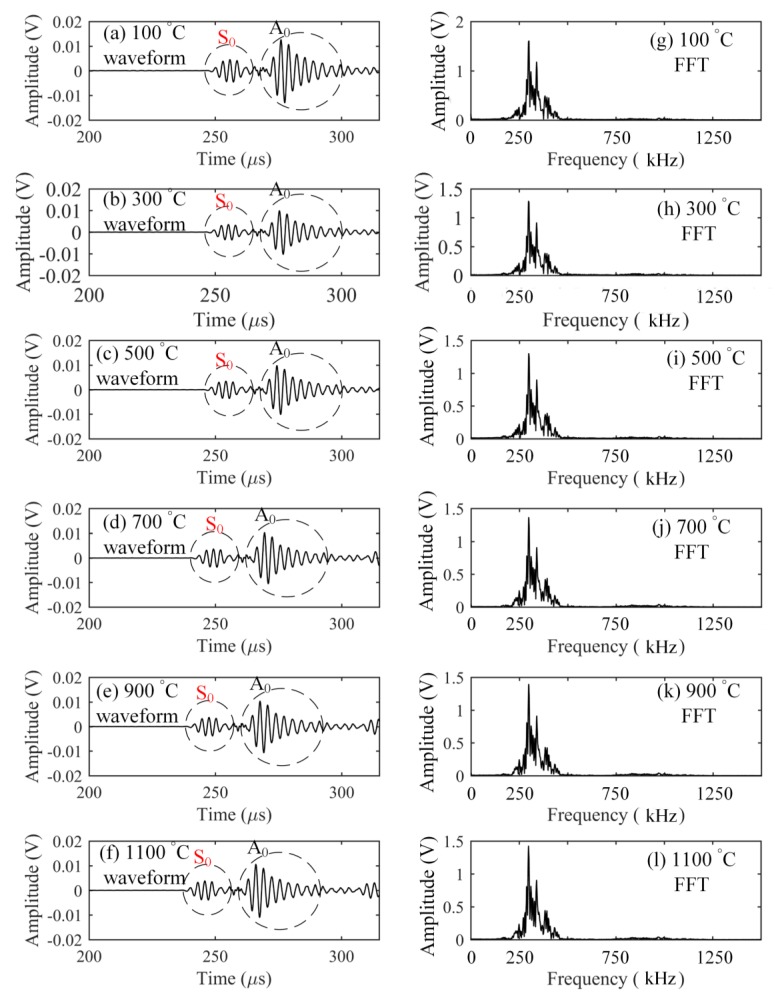
Responses to an ultrasonic wave with a center frequency of 300 kHz.

**Figure 5:**
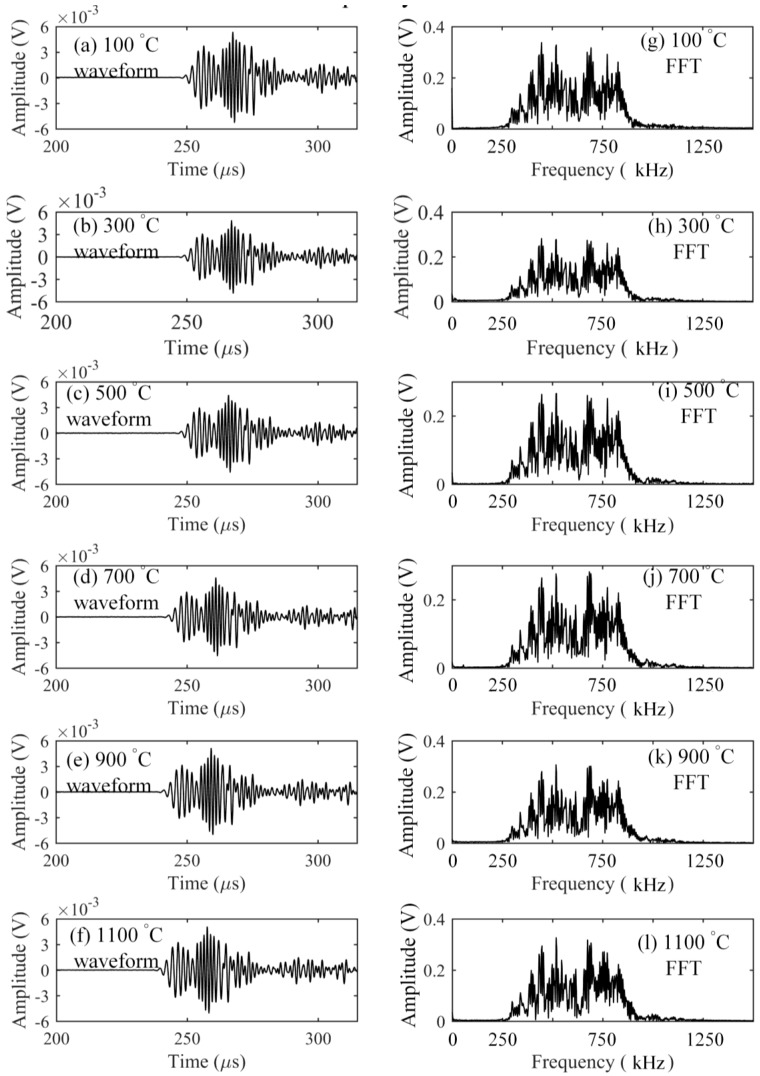
Responses to an ultrasonic wave with a center frequency of 600 kHz.

**Figure 6:**
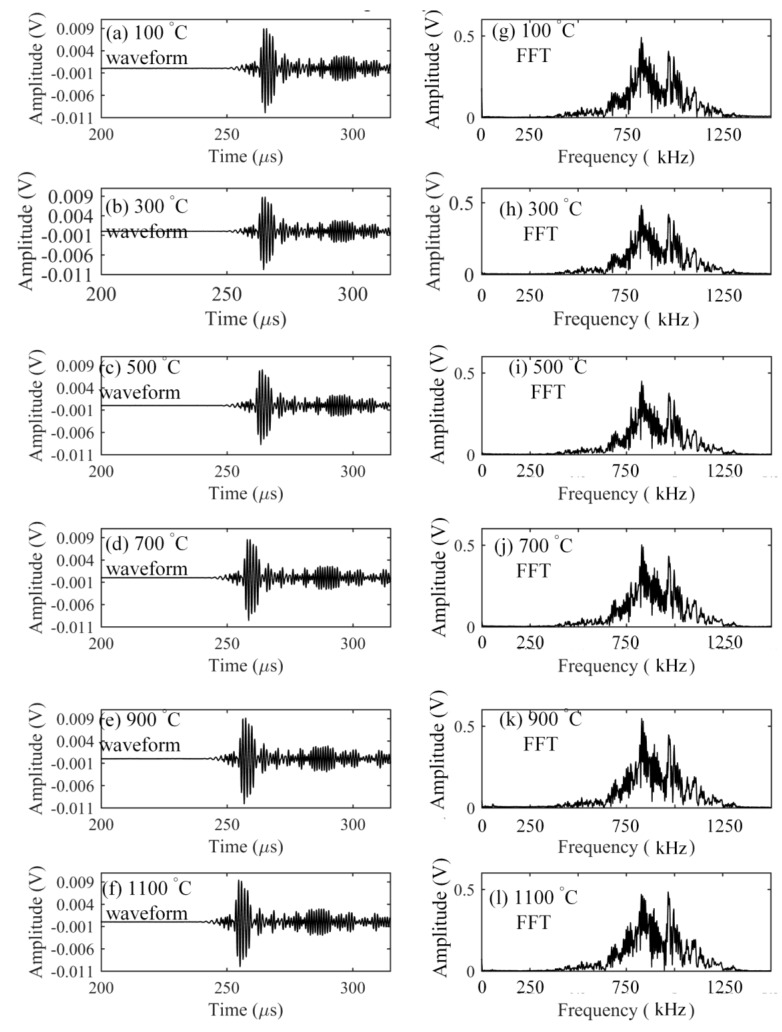
Responses to an ultrasonic wave with a center frequency of 900 kHz.

The elastic characteristics of optical fiber strongly depend on the glass transition temperature of silica [9] that is about 1185 °C [19]. Hence, to keep the stability of optical fiber-based ultrasonic waveguide, this verification experiment was conducted up to 1100 °C. Ultrasonic waves with central frequencies of 300 kHz, 600 kHz, and 900 kHz were detected at intervals of every 200 °C as the temperature was raised. First, the responses to the frequency of 300 kHz at the elevated temperatures were measured and are shown in Figure 4. On the basis of calculated theoretical dispersion curve of a Lamb wave in the aluminum plate, the S_0_ and A_0_ modes were clearly separated from the results. The figure shows that all of the detected waveforms have the same shape. The corresponding Fourier spectra at different temperatures also had the same frequency distribution. In addition, even though temperature was increased, the signal strength in the detected waveform was kept at the same voltage level. These agreements could also be observed from the results shown in Figure 5 and Figure 6 corresponding to the inputs with the central frequencies of 600 and 900 kHz, respectively. The experimental detection results indicated that the silica glass optical fiber possessed a large heat-resistance, thus enabling a stable wave propagation system without any mode transformation even at high temperatures of over 1000 °C.

**Figure 7:**
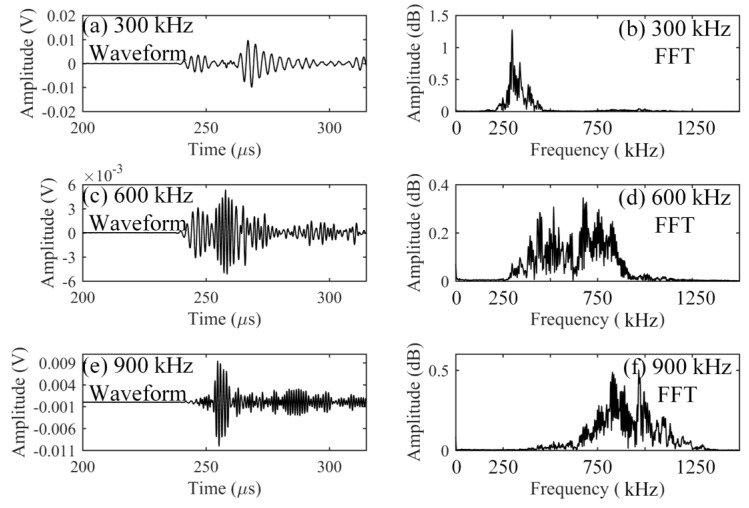
Ultrasonic detections at the temperature of 1100 °C with a duration of 8 h.

**Figure 8:**
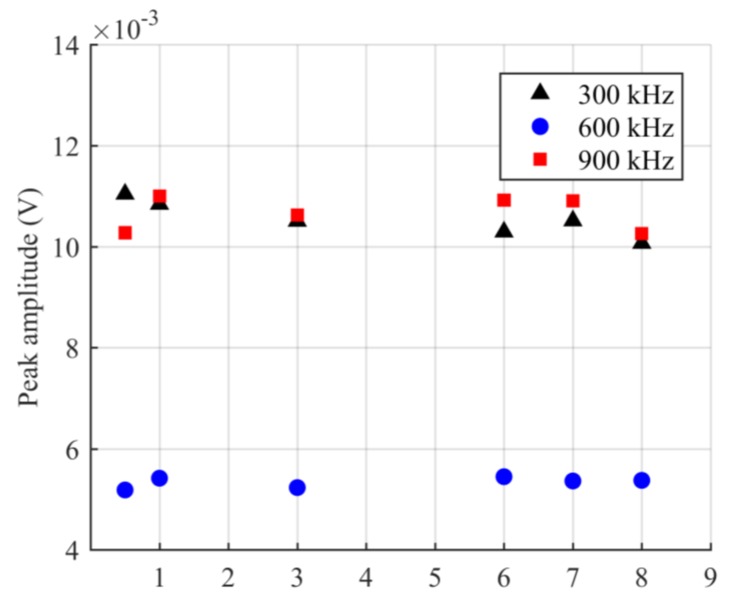
Maximum amplitude of the ultrasonic waveforms detected in the experiment to evaluate durability of the optical fiber-based waveguide at the temperature of 1100 °C.

When the ADRM configuration was used for practical NDT in some material tests, such as the high-temperature fatigue test, it was necessary to place the waveguide in a high-temperature environment for a long time. This requires a highly durable optical fiber. Therefore, we also studied the durability by keeping the optical fiber in the environment with a temperature of 1100 °C for 8 h. After 8 h, the results corresponding to the ultrasonic waves with the central frequency of 300, 600, and 900 kHz were obtained. The results in Figure 7 show the same waveform shape and spectrum as the previously obtained results presented in Figure 4, Figure 5 and Figure 6. To observe how the signal strength changed during the heating, we extracted the peak amplitudes from the detected waveform at a specific time interval for 8 h. The results were plotted as a function of the detection time in Figure 8. Examination of Figure 8 shows that the signal strengths corresponding to the individual frequency maintain the same electrical level without any damping. These results indicated that the optical fiber exhibits outstanding durability in a high-temperature environment.

Based on the experiments described above, we believe that the optical fiber can yield a highly reliable waveguide system for the remote sensing configuration in an extreme environment due to its high heat resistance.

## 3. Adhesive Point in the ADRM Configuration

In the above experiment, the cyanoacrylate-based adhesive was used to glue the optical fiber. Our previous research [18] has shown that the cyanoacrylate adhesive as room-temperature adhesive does not influence the sensing characteristics of the PSFBG sensor. However, applications at high temperature required a heat-resistant adhesive. Here, different adhesive materials gave rise to the changes in the elastic modulus at the adhesive point in the ADRM configuration, and, in turn, affected the acoustic impedance of the coupling between the plate and the optical fiber. As a result, the adhesive may influence the remote response of the PSFBG sensor to ultrasonic and AE waves.

**Figure 9:**
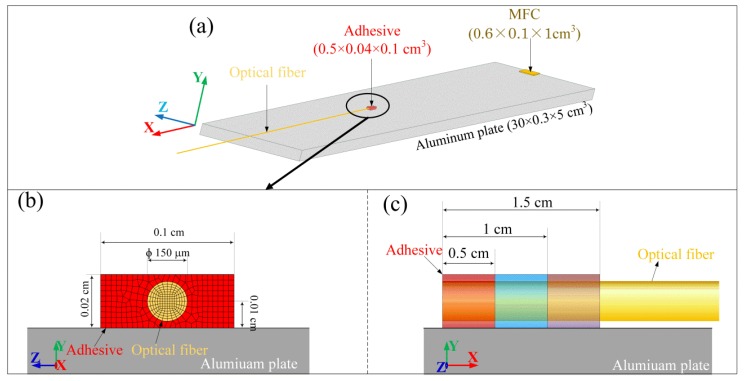
(**a**) 3-D FEM model; (**b**) model corresponding to the adhesive point; and (**c**) model for examining the influence caused by different adhesive lengths.

The coupling system consisted of two interfaces between the three system components, including plate, adhesive and optical fiber, especially those with different shapes from each other. Therefore, it was difficult to elucidate the behavior of the complex propagation system by using wave theories. Hence, the finite element model (FEM) shown in Figure 9 was used to simulate the behavior of the ultrasonic wave propagating in the ADRM configuration to examine the above-mentioned issue. A model corresponding to a piezoactuator-type macrofiber composite (MFC), a thin-film PZT-type actuator, was constructed to excite ultrasonic waves in the aluminum plate. The adhesive point was located at a distance of 20 cm from the actuator on the surface of the plate. The optical fiber was modeled as a thin silica glass cylinder with a diameter of 150 µm and embedded in the adhesive. Other details, including the element size and physical characteristics of the materials, were listed in our previous paper [18]. In particular, we note that the axial dynamic strain in the elements on the neutral axis of the fiber model corresponds to the experimental ultrasonic response of the PSFBG sensor. In the present work, outputs corresponding to detection using the PSFBG sensor in the ADRM configuration were obtained from the solid element located in the cylinder at a distance of 10 cm from the center of the adhesive point.

**Figure 10:**
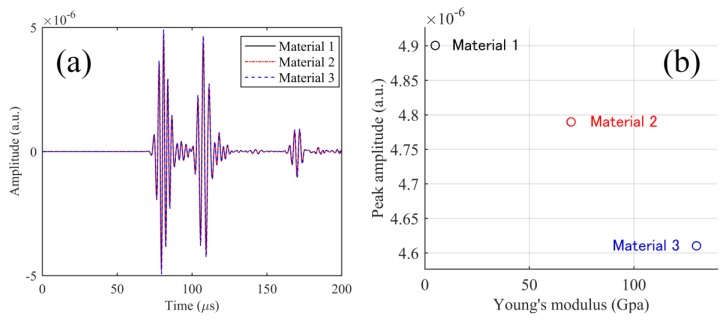
(**a**) Calculation results of the received waves in PSFBG, corresponding to the three kinds of adhesive materials listed in Table 1; and (**b**) peak amplitudes obtained from the simulation results plotted against Young’s modulus values of the adhesive materials.

Since the evaluation of the physical properties of adhesives were experimentally difficult, instead of using the values for the actual adhesive, we assigned Young’s modulus and density (Table 1) of epoxy (Material 1), aluminum (Material 2), and unidirectional CFRP (along the fiber direction) (Material 3) to the model corresponding to the adhesive point in the ADRM configuration. The changes in the acoustic impedance among the three models were evident in the large difference between the three sets of parameters. Using the three adhesive models with a length of 0.5 cm, we calculated the responses to an ultrasonic wave with a central frequency of 300 kHz. For comparison, the obtained results were shown together in Figure 10a. The figure shows that the waveforms, including the S_0_ and A_0_ modes, preserve their shapes even though Young’s modulus showed large changes. Figure 10b shows the peak amplitude extracted from the simulated waveforms to evaluate the influence of the changing elastic properties on signal strength. However, compared to the substantially increased Young’s modulus, the peak amplitudes show only very slight decreases. Moreover, it is impossible for real adhesive materials to have such large differences in the elastic properties as those between the three types of material used. Hence, we believe that a change of the adhesive material exerts a very limited influence on the AE signal detected by the ADRM configuration.

The above simulation was based on the assumption that the adhesive was homogeneous. However, we also found that porosity was generated in some high-temperature adhesives during their curing process. We note that it is better to use cements with low porosity for the practical application of the ADRM configuration, because otherwise the AE signal strength is largely decreased at the adhesive point.

In addition to the elastic properties of the adhesive material, precise control of the adhesive lengths was also difficult to achieve in practical applications. In particular, a change in temperature may deform the initial shape of the adhesive, which possibly caused an undesirable deformation in the detected waveform. Hence, as shown in Figure 9c, the lengths of the adhesive in the model were increased from 5 mm to 10 and 15 mm along the axial direction of the optical fiber (thin cylinder model) to examine how the length of the adhesive affected ultrasonic wave propagation. When increasing the length, we kept the front edge of the adhesive model at the same distance away from the actuator. This actually increased the distance between the actuator and the center of the adhesive point. The material properties of the epoxy (Material 1 in Table 1) were assigned to the model adhesive.

**Figure 11:**
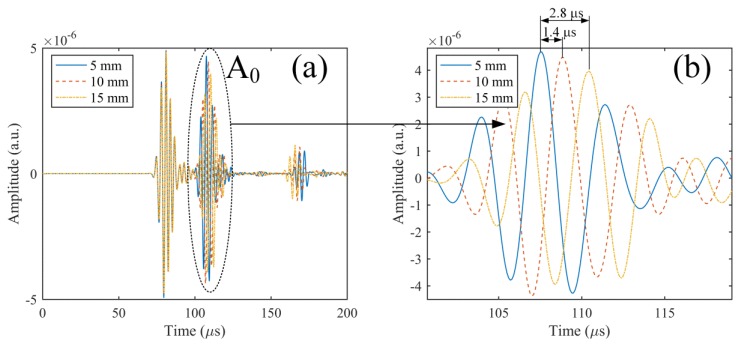
(**a**) Calculation results corresponding to the adhesive lengths of 5, 10, and 15 mm; and (**b**) magnification of the waveform components corresponding to A_0_ mode.

The responses to the same ultrasonic input as that used in the previous simulation were obtained for the three length conditions. For a clear comparison, the simulation results for the three lengths are presented together in Figure 11a,b. The results show that the increase of the adhesive length delays the arrival time of the wave components corresponding to A_0_ mode. This effect is also found for the S_0_ mode, but is too slight to be directly observed from Figure 11a. The amplitudes of the A_0_ and S_0_ modes also changed with the different adhesive length conditions. These phenomena were observed because the increase of the adhesive length moved the center of the adhesive point away from the actuator, i.e., the Lamb wave propagated for a longer distance before being guided into the optical fiber. Consequently, the results were affected by the dispersion. However, the results indicated that the change in length had no noticeable effect on the identity of the Lamb wave modes included in the simulated waveform. For instance, compared to the adhesive with a length of 5 mm, the delays in arrival time of the A_0_ mode for lengths of 10 and 15 mm (corresponding to the increase by factors of 2 and 3) were only 1.4 µs and 2.8 µs, respectively. Hence, the modes could be separated clearly from the ultrasonic signal, and the AE signal was detected using the ADRM configuration, as long as the adhesives did not show extreme deformation in a high-temperature environment. This finding was also an important sensing characteristic for the development of effective NDT methods, because analysis of the Lamb wave modes included in the AE signal has been shown to be a reliable approach for identifying the corresponding damage types in the CFRP composites [17,20,21,22].

## 4. AE Detection at 1000 °C

**Figure 12:**
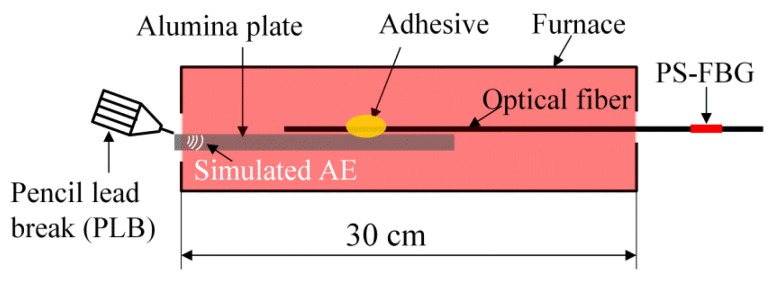
Experimental setup for detecting the simulated AE signals generated using PLB.

**Figure 13:**
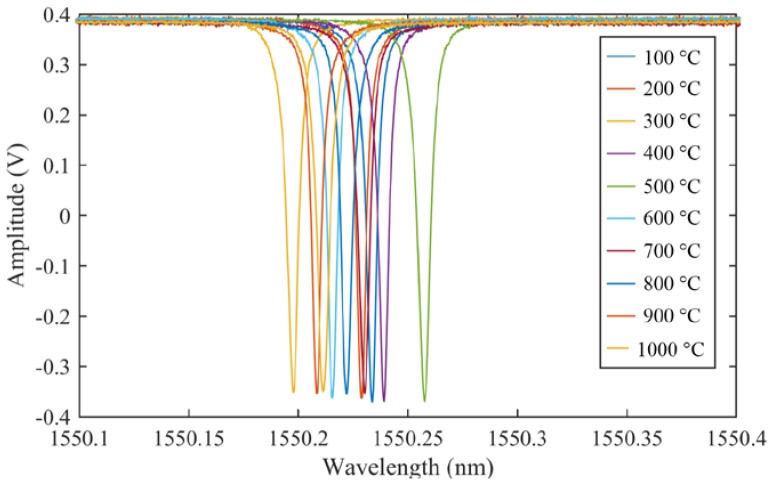
Spectra obtained by PSFBG in a high-temperature environment.

Based on the above basic research, the experiment shown in Figure 12 confirmed that the PSFBG sensor in the remote measurement configuration was able to detect the AE signal in the high-temperature environment. In this experiment, the optical fiber was bonded on the surface of a heat-resistant alumina plate by a high-temperature carbon paste (G7716, Ted Pella, Inc., Redding, CA, USA.). The cement used here shows high heat-resistance over 1500 °C and could also perform the same function in the ADRM configuration as that performed by the cyanoacrylate-based adhesive. The adhesive point was placed in the furnace. The optical fiber extended across the high-temperature environment to the room-temperature environment, where the PSFBG sensor was located and was used to remotely detect pencil lead break (PLB)-simulated AE signals. The edge condition in the reflection spectrum of the PSFBG determines the performance of the sensor in AE detection [13]. Hence, while raising the temperature at elevated temperatures up to 1000 °C, we first confirmed the spectrum by sweeping tunable laser from 1550 to 1550.5 nm with a tuning speed of 500 pm/s. Since the balanced sensing system was used to demodulate the dynamic strain [13], the spectra of the PSFBG shown in Figure 13 were also detected in the balanced conditions. The results show that the peaks of PSFBG slightly shift corresponding to temperature changes in the surrounding room temperature environment. However, the rising temperature in high-temperature furnace did not deform the shapes of the spectra. This result indicated that the ADRM configuration protected the PSFBG sensor from the degradation by heating. 

**Figure 14:**
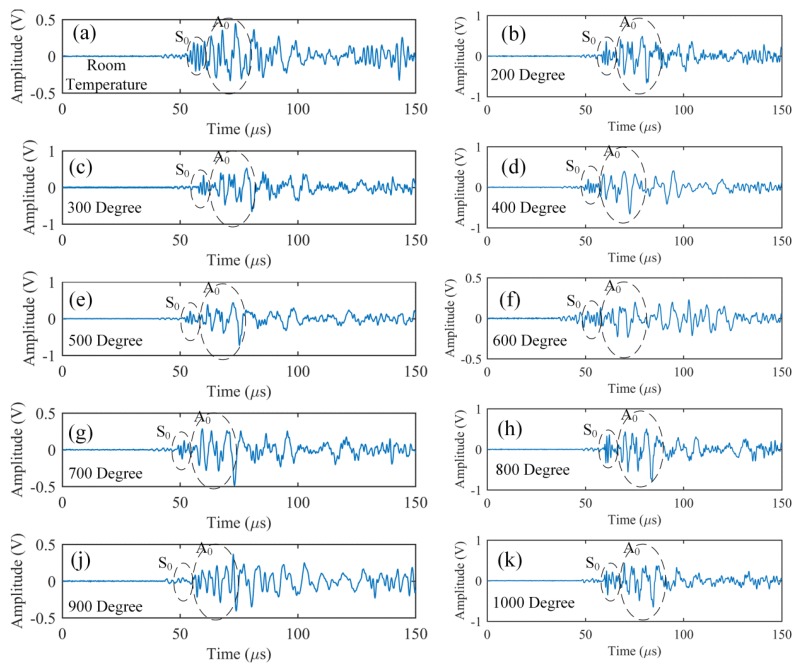
Responses of PSFBG sensor in the ADRM configuration to the simulated AEs at different high temperatures.

Finally, while raising temperature in the furnace from 100 °C to 1000 °C, we detected the simulated AE signals at every 100 °C. The obtained waveforms are presented in Figure 14 and show that the PSFBG sensor in the remote measurement configuration is suitable for AE detection at high temperatures. In particular, comparison of the results at different temperatures indicated that the rising temperature did not change the signal strength largely. Although dispersion in the plate may have added some fluctuations to the detected waveform at the elevated temperatures, the S_0_ and A_0_ modes could still be separated clearly in the results. These phenomena indicated that the remote AE measurement using the PSFBG sensor was very stable in a high-temperature environment. As mentioned above, the good performance for AE detection was mainly due to the stable waveguide system provided by the heat-resistant optical fiber under high-temperature environment. In addition, the adhesive point also exerted a negligible influence on the propagation behavior of the Lamb wave modes.

## 5. Conclusions

By taking advantage of the sensing characteristics of the PSFBG sensor in the ADRM configuration, the present work successfully achieved AE measurements at high temperatures of 1000 °C. Compared to the high-temperature detection based on PZT sensors, the remote AE measurement using the PSFBG sensor showed an outstanding performance with a high sensitivity over a broad frequency bandwidth. This was mainly due to the use of an optical fiber with high heat resistance that was able to yield a stable waveguide system for propagating ultrasonic waves even under temperatures exceeding 1000 °C. In addition, as noted in our previous research, the PSFBG sensor in the ADRM configuration responded to an original ultrasonic or AE wave at the adhesive point. As a result, the behavior of the Lamb wave modes, including the A_0_ and S_0_ modes, could be observed clearly from both the detected ultrasonic waveform and the AE waveform at high temperature. Hence, future research using the ADRM configuration-based AE detection method will potentially contribute to establishing effective NDT methods for the evaluation of microscopic damage in CMCs in engine-operating environments.

## Figures and Tables

**Table 1:** Material properties used for the adhesive model.

	Material 1	Material 2	Material 3
Density (kg m^−3^)	1146	2700	1146
Young’s modulus (Gpa)	4.83	70	130
